# Supplementary material for: Race-based biases in psychological distress and treatment judgments
Source: PLoS One. 2023 Oct 19;18(10):e0293078. doi: 10.1371/journal.pone.0293078 (PMC10586605; doi:10.1371/journal.pone.0293078)
Supplement: S1 File — (DOCX) [file pone.0293078.s001.docx]

**Analyses with Full Sample Study 1**

A paired-samples t-test assessed the primary hypothesis that participants would believe psychopathology harms Black individuals less than White individuals. As hypothesized, when paired with the same forms of psychopathology, Black target individuals were judged to experience less distress (*M*=4.55, *SD*=1.29) than White target individuals (*M*=4.67, *SD*=1.24), *t*(192)=-4.025, *p*<.001, 95% CI[-.18, -.063], *d*=0.27

As an exploratory analysis, we next tested whether responses were moderated by professional background. To test this hypothesis, we conducted a mixed-model Analysis of Variance (ANOVA) where mental healthcare experience (yes/no) was entered as a between-subjects factor and ratings of psychological distress for targets (Black/White) were entered as a within-subjects factor. Sensitivity analysis revealed this test had 80% power (α=.05) to detect small-to-medium effects (*d*=.40). Results of this analysis reproduced the previously documented main effect of target race, *F*(1, 201)=9.50, *p*=.002, and produced a main effect of professional status, such that mental healthcare professionals expected target individuals to experience more distress (*M*=5.04, *SE*=.15) than members of the public (*M=*4.39, *SE=*.10), *F*(1, 201)=12.84, *p*<.001. However, the interaction between mental healthcare experience and target race was not significant, *F*(1, 201)=.83, *p*=.363.

**Analysis with Full Sample Study 2a**

Paired samples t-tests were performed to compare judgments of Black and White targets’ psychological distress and treatment needs. Akin to Study 1, participants again judged Black targets (*M*=4.19, *SD*=1.02) to feel less psychological distress than White targets (*M*=4.33, *SD*=.93), *t*(194)=-5.22, *p*<.001, 95% CI[-.20, -.08], *d*=.37 Participants also believed that compared to White targets(*M*=12.02; *SD*=4.56), Black targets (*M*=11.39, *SD*=4.75) would need less treatment to manage their mental health, *t*(194)=-6.64, *p*<.001, 95% CI[-.82, -.44], *d*=0.47.

Using the MEMORE macro to test within-subjects mediation (Montoya & Hayes, 2017), we next tested whether race’s effect on treatment judgments was accounted for by judgments of psychological distress. Results of this mediation analysis revealed the effect of target race on treatment judgments was mediated by racial biases in distress judgments, *b*=-.37, *SE*=.08, 95% CI[-.52, -.22].

**Analyses with Full Sample Study 2b**

We first tested for racial differences in psychological distress and treatment with paired samples t-tests. Replicating the effects of Study 2a, participants judged Black targets (*M*=4.50; *SD*=1.12) to experience less distress than White targets (*M*=4.54; *SD*=1.11), *t*(275)=-1.97, *p*=.05, 95% CI[-.08, .00] *d*=0.11 and expected Black targets (*M*=10.62; *SD*=5.10) to need fewer treatment resources than White targets (*M*=10.85; *SD*=5.21), *t*(275)=-2.92, *p*=.004, 95% CI[-.38, -.07] *d*=0.16.

We next tested whether judgments of psychological distress mediated race’s effect on treatment judgments. Using the MEMORE macro (Montoya & Hayes, 2017), results indicated that target race’s effect on treatment judgments was mediated by racial biases in psychological distress, *b*=-.04, *SE*=.01, 95% CI[-.07, -.01]. Participants believed Black targets would experience less psychological distress and consequently require fewer treatment resources than White targets.

**Analyses with Full Sample Study 3**

As outlined in our pre-registration, we conducted a mixed-model ANOVA where participant background (public/MHP) was entered as a between-subjects factor and distress judgments corresponding to target race (Black/White) were entered as a within-subjects factor. Results of this analysis yielded a significant main effect of target race, *F*(1, 413)=11.59, *p*=.001, $\eta_{p}^{2}$=.027, such that Black targets (*M*=4.34, *SD*=1.31) were judged to experience less psychological distress than White targets (*M*=4.41, *SD*=1.27). There was also a main effect of participant background, *F*(1, 413)=7.86, *p*=.005, $\eta_{p}^{2}$=.019, such that MHPs (*M*=4.24, *SE*=.08) expected targets would experience more distress than lay participants (*M*=4.46, *SE*=.10). The interaction between target race and participant background was not significant, *F*(1, 413)=.17, *p*=.68.

Turning to treatment judgments, we again conducted a mixed-model ANOVA with participant background (public/MHP) as a between-subjects factor and target race as a within-subjects factor. Results of this analysis produced significant main effects of target race, *F*(1, 413)=11.39, *p*=.001, $\eta_{p}^{2}$=.027, such that Black targets (*M*=11.74, *SE=.*35) were judged to need less treatment than White targets (*M*=11.99, *SE*=.35) and participant background *F*(1, 413)=18.27, *p*<.001, $\eta_{p}^{2}$=.042, such that MHPs (*M*=13.34, *SE*=.54) made greater treatment judgments than members of the public (*M*=10.40, *SD*=.43). The interaction between participant background and target race did not reach significance, *F*(1, 413)=3.25, *p*=.072, $\eta_{p}^{2}$=.008.

To test whether target race had an indirect effect on treatment judgments via racial biases in psychological distress, we used the MEMORE macro (Montoya & Hayes, 2017) to test race’s effect on treatment judgments through distress judgments among members of the public. For lay participants, the effect of target race on treatment judgments was mediated by racial biases in distress judgments, *b*=-.19, *SE*=.074, 95% CI [-.35, -.06].
